# Supplementary material for: Every day mitral valve reconstruction: What has changed over the last 15 years?
Source: PLoS One. 2022 Oct 3;17(10):e0269537. doi: 10.1371/journal.pone.0269537 (PMC9529113; doi:10.1371/journal.pone.0269537)
Supplement: Attachment — Submitted filename: Response to Reviewers.docx [file pone.0269537.s001.docx]

**Response to Reviewers**

EVERY DAY MITRAL VALVE RECONSTRUCTION: WHAT HAS CHANGED OVER THE LAST 15 YEARS?

Dear Reviewers,

Thank you for your thoughtful comments and suggestions, which we would like to respond to as follows:

Reviewer #1:

Mitral valve pathologies are indeed important. I am sure you know from your own experience that it is somewhat problematic to determine the exact underlying pathology in a retrospective study with an almost historical cohort going back to 2004, with few meaningful echocardiographic findings properly documented or with not much in the surgeon's protocol or in the letter from the referring hospital. Thus, pathologies were not the primary focus of interest in this retrospective study. However, on your advice, we reviewed all the data still available and obtained as much information as possible. In the end, we were able to distinguish quite well between primary and secondary nature of the origin of mitral valve regurgitation by considering the morphology as primary origin if the valve leaflets and other aspects directly affected the valve apparatus or as secondary origin if the valves were tethered and/or the annulus was asymmetrically distorted. In the remaining cases, whose pathological origin was still unclear, we assumed that it was a secondary condition if bypass surgery was performed at the same time. Indeed, our results show differences between patients with measures on the valves themselves (resection, reconstruction, repair) and those with concomitant bypass surgery. In this way, we were able to investigate, at least indirectly, the importance of the type of mitral valve regurgitation (primary or secondary origin) on outcome. We included mitral valve pathologies in Table 1 and referred to them in the Patients and Methods section (lines 72-5) and in the Results (line 107), Discussion (245-9), and Limitations (lines 291-3). We also cited the 2021 ESC guidelines, which contain the current mitral valve pathology systematics [5].

Reviewer #2:

1. The slight decrease between period 2 and 3 was indeed apparent. After noticing a steep increase in repair from period 1 to period 2 possibly owing to the experience gained with repair strategies in the early years in our institution a slight decrease of the proportion of repair maneuvers was noted declining from 48% to 41%. This shift was not due to a more restrictive policy regarding repair surgery but was instead non-intentional and can be explained by a higher morbidity of the patients resulting in a higher proportion of patients with complex redo-surgery or endocarditis. We explained the slight decrease of the proportion of mitral valve repair and added a respective statement in the Discussion (lines 239-44).
2. Only 13 minimally invasive mitral valve repairs were performed mainly in period 2. The true minimally invasive approach was not favored in our hospital until recently because of the lack of center-wide expertise performed by multiple surgeons. So, you are correct. This was a center decision at the time, which has now been changed after several experienced surgeons had been trained in the meantime with minimally invasive mitral valve repair with left mini-thoracotomy at other institutions with high experience and volume. We added a respective statement in the discussion (lines 250-2)
3. We have erased the term MIC in tables, legends, and figure 2 and replaced it by MIS-MVR.
4. We added respective literature evidence regarding our statement: These particular changes comprised wider rings with multi-layer fabric to allow for better stitching and more secure anchoring. Stiff or moderately flexible rings appeared to stabilize the annulus significantly better than highly flexible ones [10,11].

Thank you again for your valuable comments.

Johannes M. Albes, MD, PhD, MBA
